# DKK1 promotes hepatocellular carcinoma inflammation, migration and invasion: Implication of TGF-β1

**DOI:** 10.1371/journal.pone.0223252

**Published:** 2019-09-30

**Authors:** Maha Fezza, Mayssam Moussa, Rita Aoun, Rita Haber, George Hilal

**Affiliations:** Cancer and Metabolism Laboratory, Faculty of Medicine, Saint-Joseph University, Beirut, Lebanon; National Cancer Center, JAPAN

## Abstract

Dickkopf-1 (DKK1), an inhibitor of the most frequently impaired signaling pathway in hepatocellular carcinoma (HCC), the Wnt/beta-catenin pathway, seems to fulfill contradictory functions in the process of tumorigenesis, acting either as an oncogenic promoter of metastasis or as a tumor suppressor. Elevated serum levels of DKK1 have been reported in HCC; however, little is known about its functional significance. In the current study, we treated HepG2/C3A and PLC/PRF/5 with the recombinant protein DKK1. Cytotoxicity was first determined by the WST-8 assay. AFP expression was measured at both the mRNA and protein levels. Expression of the oncogenes *MYC*, *CCND1*, *hTERT*, and *MDM2* and the tumor suppressor genes *TP53*, *P21* and *RB* was assessed. Western blot analysis of non-phosphorylated ẞ-catenin and Sanger sequencing were performed to explain the functional differences between the two cell lines. Subsequently, inflammation, migration and invasion were evaluated by qPCR, ELISA, the Boyden chamber assay, zymography, and MMP-2 and MMP-9 western blot analysis. Knockdown of DKK1 and TGF-β1 were also performed. Our results suggest that DKK1 exerts an oncogenic effect on HepG2/C3A cell line by upregulating the expression of oncogenes and downregulating that of tumor suppressor genes, whereas the opposite effect was demonstrated in PLC/PRF/5 cells. This differential impact of DKK1 can be explained by the mutations that affect the canonical Wnt pathway that were detected in exon 3 of the *CTNNB1* gene in the HepG2 cell line. We further confirmed that DKK1 promotes inflammation, tumor invasion and migration in both cell types. The canonical pathway was not responsible for the DKK1 proinvasive effect, as indicated by the active ẞ-catenin levels in the two cell lines upon DKK1 treatment. Interestingly, knockdown of TGF-β1 negatively affected the DKK1 proinvasive effect. Taken together, DKK1 appears to facilitate tumor invasion and migration through TGF- β1 by remodeling the tumor microenvironment and inducing inflammation. This finding endorses the relevance of TGF-β1 as a therapeutic target.

## Introduction

GLOBOCAN estimates that in 2018, approximately 841,000 new cases of liver cancer and 782,000 related deaths were reported, marking liver cancer as the sixth most commonly diagnosed cancer and the fourth leading cause of cancer-related death worldwide [1]. Hepatocellular carcinoma (HCC) is the most prevalent liver neoplasm, comprising 75 to 85% of all cases [1]. HCC usually occurs in the setting of cirrhosis resulting from different etiological factors (i.e., chronic alcohol consumption, chronic hepatitis B (HBV) and C (HCV) viral infection, and obesity, among others). The incidence of HCC is steadily increasing in areas with historically low rates, owing to the increase in HCV-associated liver cirrhosis and a high prevalence of non-alcoholic steatohepatitis [2].

Despite the recent advances in diagnosis and treatment, the prognosis of advanced HCC remains poor, with an estimated 5-year survival rate [3]. In fact, fewer than 20% of patients are considered eligible for curative treatment modalities due to a late diagnosis that is established when the tumor has already invaded the liver parenchyma, spread through the portal venous system, or formed metastases [4]. In this context, elucidating the molecular basis governing the progression and metastasis of HCC is crucial for the development of new diagnostic and therapeutic tools to improve the prognosis. To date, the precise molecular mechanisms underlying hepatocarcinogenesis are not fully understood [5]. However, it is well established that the development of HCC is a complex multistep biological process resulting from the progressive accumulation of genetic and epigenetic alterations that affect progenitor cells or terminally differentiated hepatocytes, allowing them to acquire stemness features [6,7]. While most genetic alterations occur in genes with no known carcinogenic consequences, a small number of these alterations occur in pilot genes that are members of key signaling pathways involved in carcinogenesis [8].

Exome sequencing approaches have revealed that 11 pathways are altered in more than 5% of HCC cases, and the WNT/β-Catenin pathway is aberrantly activated in 54% of cases, making this pathway by far the most frequently altered oncogenic pathway in HCC [9]. ẞ-catenin, a key intracellular transducer of this pathway, is recurrently mutated in human cancers [10]. In fact, the ẞ-catenin gene can acquire oncogenic activity as the result of activating mutations that lead to the loss of the phosphorylation consensus sequences involved in the negative regulation of ẞ-catenin by the APC/AXIN1/GSK3 destruction complex [11]. These mutations induce aberrant activation of Wnt signaling and reprogramming of downstream nuclear networks and have been associated with tumor progression and a poor prognosis [12].

The Wnt signaling pathway diversifies into two major signaling cascades: a canonical or β-catenin-dependent pathway and a noncanonical or β-catenin-independent pathway. In the canonical pathway, signaling begins with the binding of the Wnt ligand to its corresponding receptor complex, which is made up of Frizzled (Fz) and low-density lipoprotein receptor-related protein 5/6 (LRP5/6), leading to the dissolution of the complex that is required for β-catenin destruction. This dissolution results in the accumulation of active β-catenin in the cytoplasm and eventually induces its translocation to the nucleus, where it binds to members of the TCF/LEF family of transcription factors and triggers the transcription of genes involved in carcinogenesis, such as *CCND1* and *MYC* [13,14]. Disrupting the activation of Wnt signaling may therefore be a promising therapeutic strategy for treating HCC.

Dickkopf1 (DKK1), which is a negative regulator of the Wnt/beta-catenin pathway, is a soluble secreted protein with a low molecular weight. DKK1 is involved in head formation during embryogenesis, is weakly expressed in adult tissues, and has an inhibitory effect on the Wnt/β-catenin pathway [15]. DKK1 binds competitively to LRP 5/6, for which it has a higher affinity compared with the Wnt ligands, thereby preventing the formation of the Fz-Wnt-LRP5/6 complex and intercepting Wnt signal transduction [16].

DKK1 seems to fulfill very contradictory functions in the tumorigenesis process, acting either as an oncogenic promoter of metastasis or as a tumor suppressor, depending on the cell type and context [17]. Qi L et al. suggested an inverse correlation between the expression of DKK1, the tumor stage and the presence of metastases in colon cancer; this group demonstrated that the antitumor effect of DKK1 is related to its ability to inhibit the epithelial-mesenchymal transition (EMT) [18]. Another study in the same context revealed that DKK1 expression is decreased in human colon tumors, indicating that this molecule acts as a tumor suppressor by inhibiting the expression of target genes of the beta-catenin-TCF pair [19].

Similarly, a significant decrease in DKK1 expression was observed in renal cancer tissues, and a tumor suppressor effect was demonstrated following the transfection of renal cancer cell lines with recombinant plasmids containing the DKK1 gene [20]. On the other hand, Jing Zhang et al. designated DKK1 as an oncogene that promotes the invasion and migration of non-small-cell lung cancer by inhibiting the phosphorylation of beta-catenin [21]. Other recent studies showed that DKK1 is a serological marker of breast cancer metastasis organotropism, suppressing lung metastasis but promoting bone metastasis of breast cancer [22,23].

DKK1 overexpression has been reported in hepatoblastoma, suggesting that DKK1 acts as an oncogenic factor through inhibition of the Wnt signaling pathway [24]. However, the role of DKK1 in HCC is still poorly understood. A large-scale multicenter study, published in Lancet Oncology, has shown that DKK1 is overexpressed in HCC and that the levels of DKK1 in serum can be used in addition to AFP levels to improve the screening of AFP-negative HCCs and to distinguish them from nonmalignant chronic liver diseases; however, more studies are needed before DKK1 can be accepted as a diagnostic tool for HCC [16,25]. Moreover, the downregulation of DKK1 using siRNA was shown to inhibit invasion and metastasis in the HCC cell line HCCLM3, which is known for its high metastatic potential. In contrast, the overexpression of DKK1 in the HepG2 cell line (with low metastasis potential) significantly promoted migration and invasiveness [26]. Qin et al. investigated the role of DKK1 in the proliferation and migration of HCC cells and reported that the overexpression of DKK1 by transfection inhibited growth and migration of the M-H7402 cell line [27]. However, the direct effect of recombinant DKK1 on the tumor microenvironment of HCC has not yet been documented. Our results indicate that recombinant DKK1 has a proinvasive effect that is associated with an exacerbation of the inflammatory process in both the HepG2/C3A and PLC/PRF/5 hepatocellular carcinoma cell lines. This common effect of DKK1 appears to be related to the increase in the expression of the proangiogenic factor TGF-β. Regarding the effect of DKK1 on oncogenes and tumor suppressor genes expression, this effect appears to depend on the cell type and the underlying genetic components. Thus, DKK1 acts as a tumor suppressor in PLC/PRF/5 and as a proto-oncogene in HepG2/C3A.

## Materials and methods

### Culture and maintenance of cell lines

The human hepatocellular carcinoma cell lines HepG2/C3A and PLC/PRF/5 were purchased from the American Type Culture Collection (ATCC, Manassas, VA, USA). The PLC/PRF/5 cell line (ATCC, CRL-8024^™^) was cultured at an appropriate density in DMEM/F12 (Dulbecco’s modified Eagle’s medium, SIGMA-ALDRICH) supplemented with 10% fetal bovine serum (FBS) and 1% penicillin/streptomycin. The HepG2/C3A cell line (ATCC, CRL-10741^™^) was cultured in a low-glucose DMEM (1 g/l) supplemented with 10% FBS and 1% penicillin/streptomycin. All cells were maintained at 37 ˚C in an atmosphere of humidified air with 5% CO2. The culture of both cell types was performed in 75-cm^2^ flasks, and the medium was changed every 2 to 3 days.

### Treatment with Dickkopf-related protein 1 (DKK1)

Both cell lines (HepG2/C3A, PLC/PRF/5) were treated with a recombinant DKK1 solution with increasing DKK1 concentrations of 10, 100, 150, and 200 ng/ml.

The recombinant DKK1, provided by R&D SYSTEMS, was reconstituted at a concentration of 100 μg/ml in PBS solution (Phosphate-buffered saline) supplemented with 0.1% FBS. Then, the reconstituted protein was added to serum-free medium (DMEM/F12) to achieve the desired concentrations. The cells were exposed to DKK1 for 24, 48 or 72 hours.

### Cytotoxicity assay

The cytotoxicity of DKK1 was evaluated using a WST-8 cell counting kit according to the manufacturer’s instructions (Sigma-Aldrich, Germany). Briefly, 10^4^ cells per well were seeded in 96-well plates and incubated for 48 hours in DMEM/F12 (10% FBS). The medium was then removed and replaced with recombinant DKK1 solutions (0.10, 100, 150, 200 ng/ml). After 24, 48 and 72 hours, 10 μl of tetrazolium salt was added to each well, and the formazan formation was assessed using an ELISA reader Multiskan Go at 450 nm.

### Quantification of AFP levels by ELISA

HepG2/C3A and PLC/PRF/5 cells were subcultured into 24-well cell culture plates (5*10^4^ cells per well) and incubated for 48 hours; the complete medium was then removed and replaced with DKK1 at 10, 100, 150, and 200 ng/ml. After 24, 48 and 72 hours, the supernatant was removed and used for the AFP quantification assay, while the cells were washed with PBS, trypsinized, counted and then lysed with the addition of lysis buffer for RNA extraction.

The levels of AFP secreted in the culture medium from HepG2/C3A and PLC/PRF/5 cells were quantified using the HUMAN AFP ELISA kit (GmbH Germany) as recommended by the manufacturer. The optical density (O.D.), which is proportional to AFP concentration, was measured using an ELISA reader Multiskan Go at 450 nm.

### RNA extraction and real-time PCR

RNA was extracted from samples using the NucleoSpin® RNA extraction kit (Macherey-Nagel, USA) according to the manufacturer’s instructions. RNA quality and yields were analyzed using a NanoDrop spectrophotometer (ND-1000). The first-strand cDNA synthesis was performed using the iScript^™^ cDNA-synthesis Kit (Bio-Rad Laboratories, CA).

Quantification of gene expression was performed by real-time PCR with a QuantiFast SYBR Green Kit (QIAGEN, USA) and the primers listed below (S1 Table). cDNA was used to measure the expression levels of the following panel of genes: c-Myc, hTERT, TIMP-1, TIMP-2, TIMP-3, MMP-2, MMP-9, p53, RB, p21, MDM2, TGF-ẞ, TNF-α, IL-1ẞ, IL-6 and AFP. The relative expression of these genes was normalized to that of glyceraldehyde 3- phosphate dehydrogenase (GAPDH). Briefly, 1 μl of cDNA was brought to a concentration of 5 ng/μl and added to a mixture of 4 μl of SYBR green, 4.5 μl of nuclease-free water and 0.25 μl of each primer to obtain a final reaction volume of 10 μl, which was then introduced into the real-time cycler Rotor-Gene® Q (Qiagen, USA). The PCR thermal cycling conditions included a predenaturation step at 95°C for 5 minutes and then 40 cycles including denaturation at 95°C for 6 minutes followed by annealing and extension at 60°C for 30 sec. Melting curves were established at the end of the PCR to ensure that only one product was amplified. An amplification curve with an exponential phase was obtained, which allowed for the determination of the threshold cycle Ct. The relative expression was calculated using the value of 2^-ΔΔCt^ (Delta Ct = Ct gene—Ct endogenous control, Delta Delta Ct = ΔCt sample1 - ΔCt calibrator). The entire analysis was performed in triplicate.

### DNA extraction, amplification and sequencing

Genomic DNA was digested by a proteinase K/SDS solution then extracted with Nucleospin^®^ Tissue Columns (Macherey-Nagel, Germany) according to the manufacturer’s protocol. DNA quality and yields were analyzed using a NanoDrop spectrophotometer (ND-1000). Specific primers were designed using PRIMER3 software to amplify exon 3 and 4 and flanking intronic sequences of the *CTNNB1* gene (the primers are provided in S1 Table). PCR of genomic DNA [1× FIREPol ^®^ Master Mix, 0.3 μM forward and reverse PCR primers, 0.1 ng/μl genomic DNA] was carried out with the thermal cycling conditions of 95°C for 5 min, followed by 30 cycles of 95°C for 30 secs, 51.5°C for 1 min, 72°C for 1 min, followed by 72°C for 5 min. PCR products were visualized by electrophoresis on a 2% agarose gel with SYBR GREEN. The remaining PCR products were purified using the ExoSAP-IT^TM^ Kit (Thermo Fisher Scientific, USA) and sequenced using automated Sanger sequencing. The sequence chromatograms were analyzed using ChromasPro software with NM_001098209.1 as a reference sequence.

### ELISA Detection of TGF-β1, TNF-α and IL-6

Protein levels of TGF-β1, TNF-α and IL-6 were measured in the supernatants using sandwich ELISA kits purchased from R&D systems (Human TGF-beta 1 DuoSet, Human TNF-alpha DuoSet, Human IL-6 DuoSet ELISA). Each well of a 96-well plate was coated overnight at room temperature with mouse anti-human capture antibody. The plates were washed with TBS, 0.05% Tween 20, blocked for a minimum of 1 hour with 5% BSA (Sigma-Aldrich) in TBS and washed once more with TBST. Then, 100 μl of each sample diluted with 1% BSA in TBS was incubated for 2 hours at room temperature. Recombinant human TNF-α, TGF-β1 and IL-6 were used as standards. The plates were washed with TBST and then incubated with detection antibody for 2 hours at room temperature. The plates were washed again with TBST and incubated for 20 minutes with HRP-streptavidin at room temperature. The plates were washed once again with TBST and incubated with the substrate solution for 20 minutes. Reactions were stopped with 2N H_2_SO_4_. The optical density of the colorimetric reaction in each well was determined at 450 nm.

### Genetic knockdown

Cells were seeded in six-well plates and transfected with 40 nM of small interfering RNA (siRNA) using Hiperfect^®^ transfection reagent (Qiagen, USA) according to the manufacturer’s instructions. The oligonucleotide sequences Hs_DKK1_1 (ATGTACTATCTTAATGCTTAA) and Hs_TGFB1_6 (CAGCATATATATATGTTCTTCAA) were purchased from Qiagen. AllStars Negative Control siRNA from Qiagen was used in parallel. Knockdown was confirmed by real time PCR (S1 Fig). Cells transfected for 24 h with TGF-beta siRNA were then treated with recombinant DKK1. The supernatant was collected and concentrated using Microcon® Centrifugal Filter Devices (Merck Millipore, Ireland). The pellets were harvested for RNA and protein extraction.

### Western blotting

To investigate whether DKK1 exerts the same effect on the Wnt pathway in HepG2/C3A and PLC/PRF/5 cells, protein levels of active beta-catenin were determined by western blotting. MMP-2 and MMP-9 protein levels were also evaluated. Cells were lysed using RIPA protein extraction reagent (Sigma-Aldrich, USA) in the presence of sodium orthovanadate, a protease inhibitor cocktail and PMSF, all purchased from Sigma. The supernatant was collected and concentrated by ultracentrifugation. Protein concentrations were measured with a BCA protein-assay Kit (BIO-RAD, USA) for cell lysates and with the Bradford reagent (BIO-RAD, USA) for the concentrated supernatants. Equal amounts of protein samples were subjected to 10% SDS-PAGE analysis and electrophoretically transferred to PVDF membranes. Membranes were then blocked with 5% BSA (Sigma-Aldrich, USA) for 1 hr, then probed with primary antibodies: rabbit monoclonal antibody MMP-2 (40994), rabbit monoclonal antibody MMP-9 (13667), or rabbit monoclonal non-phospho (active) β-catenin (8814) at 4°C overnight, then incubated with HRP-linked anti-rabbit antibody at room temperature for 2 hrs. All antibodies were purchased from Cell Signaling Technology (Danvers, MA, USA). Signals were visualized using an Optiblot ECL Detect Kit (Abcam, USA).

### SDS-Gel Gelatin Zymogram

The gelatinolytic activity of the matrix metalloproteinases MMP-2 and MMP-9 was analyzed in the supernatants collected after HepG2/C3A and PLC/PRF/5 cells were treated with recombinant DKK1. A total of 30 μg of protein from each sample was separated on an 8% polyacrylamide gel containing 2 mg/ml gelatin and 0.1% SDS under nonreducing conditions with running buffer (25 mM Tris, 192 mM glycine, 0.1% SDS, pH 8.3). After electrophoresis, the gel was washed 3 times for 30 min each in a Triton X-100 solution [2.5% Triton X-100, 50 mM Tris-HCL (pH 7.5), 5 mM CaCl_2_ and 1 μM ZnCl_2_] at room temperature to remove the SDS. The gel was then incubated in developing buffer (1% Triton X-100, 50 mM Tris HCL, 5 mM CaCl_2_, 1 μM ZnCl_2_) for 24 hours at 37 ˚C; stained in a solution of 0.05% Coomassie Brilliant Blue g-250, 25% methanol and 10% acetic acid for 1 hour at room temperature; and then destained in a solution of 8% acetic acid and 4% methanol. Gelatinolytic activity was detected as clear bands against a blue background.

### Migration assay

The effect of DKK1 on cell migration was evaluated using 24-well-modified Boyden chambers designed by Cell Biolabs (USA). According to the manufacturer's instructions, 3*10^5^ HepG2/C3A cells that were treated with DKK1 were suspended in serum-free medium in the upper chamber of each well. The same medium supplemented with 10% FBS was added to the lower chamber of each well to act as a chemo-attractant solution. After 24 hours of incubation, the cells that migrated to the lower chamber of the wells were stained using a staining solution that fixes and stains the cell walls and cytoplasmic membranes of any cell remaining on the insert. The stain is instantly dissolved once the extraction solution is added. The solution was then transferred to a 96-well microtiter plate, and the absorbance was measured at 560 nm using a plate reader.

### Invasion assay

The invasion ability of HepG2/C3A cells was evaluated using 24-well-modified Boyden chambers coated with ECMs (Extracellular matrix proteins) designed by Cell Biolabs (USA). According to the manufacturer's instructions, 3*10^5^ cells in a serum-free DMEM were seeded onto the upper chamber of each well. The same medium supplemented with 10% FBS was added to the lower chamber to act as a chemo-attractant solution. After 48 hours of incubation, the cells that invaded the bottom of the membrane were stained. An extraction solution was then added, and the mixture was transferred to a 96-well microtiter plate. Finally, the cells were quantified at 560 nm using a plate reader.

### Statistical analysis

GraphPad Prism software version 7.0 was used for the statistical analysis.Data variability is expressed as the mean ± standard deviation (SD). Differences between groups were analyzed by one-way ANOVA or a t-test. Significance is denoted by *, ** and *** in the case of a *p*-value <0.05, <0.01 and <0.001, respectively.

## Results

### DKK1 treatment enhanced the viability rate and AFP mRNA expression

**Fig 1:**
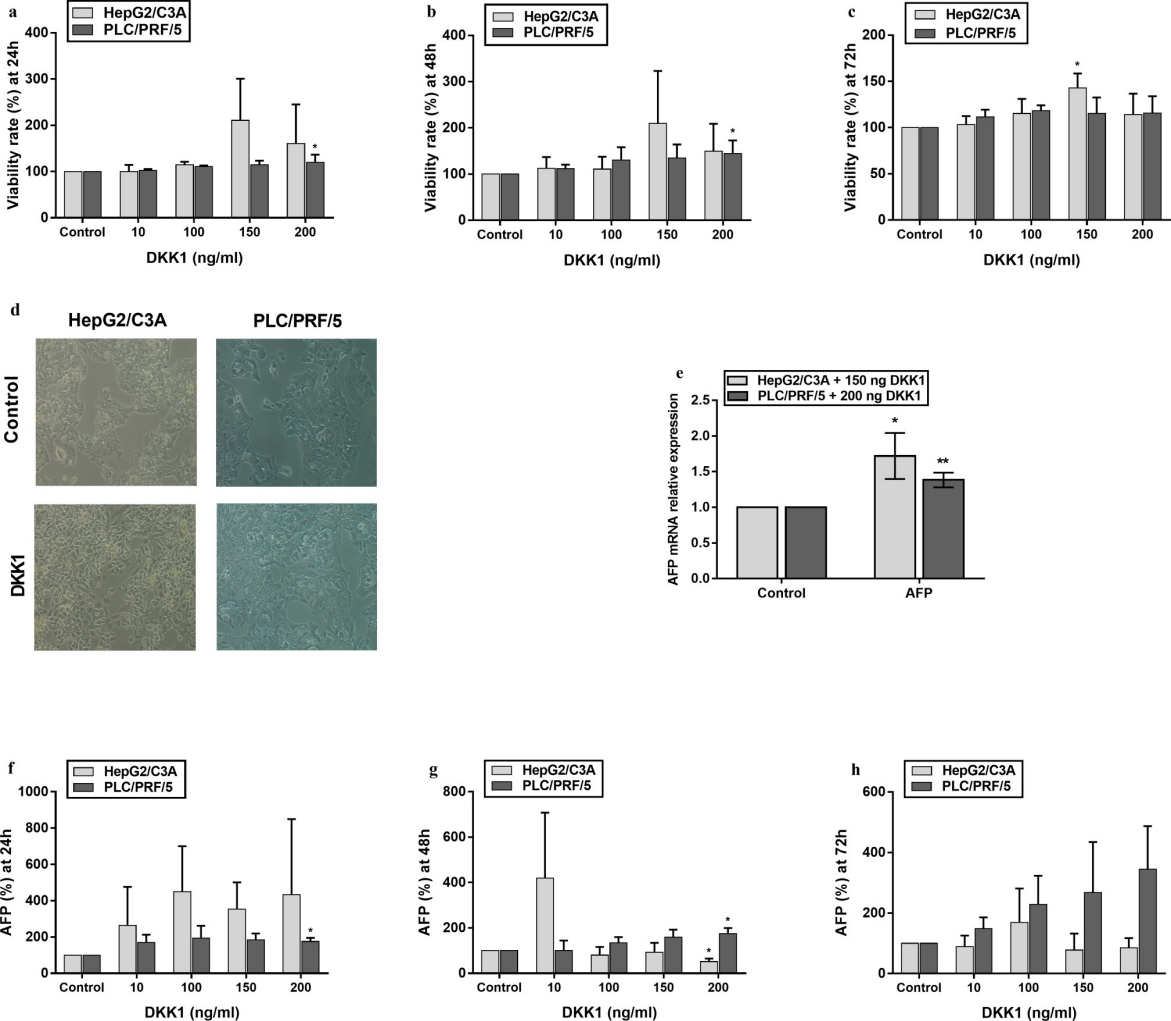
Effect of DKK1 on the viability rate and on the AFP protein and mRNA expression levels in hepatocellular carcinoma cell lines. HepG2/C3A and PLC/PRF/5 cells were seeded at a density of 10^4^ cells/well. The viability rates of HepG2/C3A and PLC/PRF/5 obtained after 24 h, 48 h or 72 h of DKK1 exposure were calculated from the absorbance values **(a, b, c)**. Experiments were performed on 5 batches of each cell line. **(d)** Representative photomicrographs showing the proliferation of HepG2/C3A and PLC/PRF/5 after DKK1 treatment. **(e)** mRNA expression levels of AFP were measured by qPCR in HepG2/C3A and PLC/PRF/5 cells after treatment with 150 ng/ml and 200 ng/ml recombinant DKK1, respectively. AFP concentration in the cell culture supernatant of HepG2/C3A and PLC/PRF/5 cells treated with different concentrations of DKK1 for 24 h **(f)**, 48 h **(g)** or 72 h **(h)**, as measured by sandwich ELISA. Data are expressed as the mean ± SD. * p <0.05, ** p <0.01, *** p <0.001, as indicated.

The cytotoxicity assay revealed that DKK1 was not cytotoxic towards hepatocellular carcinoma cell lines at any concentration tested. Fig 1A, 1B and 1C show that the viability rate of PLC/PRF/5 increased in response to increasing doses of DKK1. In fact, a significant increase of 44% (*p* < 0.05) in the cell viability rate was noticed at the concentration of 200 ng/ml at 48 hours (Fig 1B). Likewise, in HepG2/C3A cells, a significant viability increase of 45% (*p* < 0.05) at a concentration of 150 ng/ml at 72 hours was recorded (Fig 1C).

We also evaluated the effect of DKK1 on AFP production in our cell lines. AFP is used as a tumor biomarker for the diagnosis and follow-up of hepatocellular carcinoma. Recently, many studies have suggested that AFP affects HCC cell growth, apoptosis and migration [28]. AFP secretion was therefore measured using the AFP sandwich ELISA Kit. A significant increase in the production of AFP (of approximately 75%) at a concentration of 200 ng/ml at both 24 and 48 hours (Fig 1F and 1G) was observed for the PLC/PRF/5 cell line. In the HepG2/C3A cell line, a significant decrease was observed at a concentration of 200 ng/ml at 48 hours (Fig 1G). Knowing that the HepG2/C3A doubling time is approximately 48 hrs and that treatment was performed with cells between 60 and 85% confluency; the decrease observed at 48 hrs cannot be attributed to DKK1 treatment as, according to the ATCC, a marked reduction occurs in AFP secretion by the HepG2/C3A cell line as the cells become confluent, which was likely the case at 48 hrs of treatment. Later, a qPCR assay was performed. The results (Fig 1E) revealed that AFP transcription was significantly increased (1.72-fold) in the HepG2/C3A cells (*p* < 0.05). The same situation occurred in the PLC/PRF/5 cell line with a very significant increase (1.38-fold) (*p* < 0.01). DKK1 treatments with 150 ng/ml for 72 hours and 200 ng/ml for 48 hours were selected as the optimal concentrations and durations for HepG2/C3A and PLC/PRF/5, respectively, for the following experiments.

### DKK1 induces differential effects on the Wnt/beta-catenin pathway and on its target genes as a result of mutations in the *CTNNB1* gene

**Fig 2:**
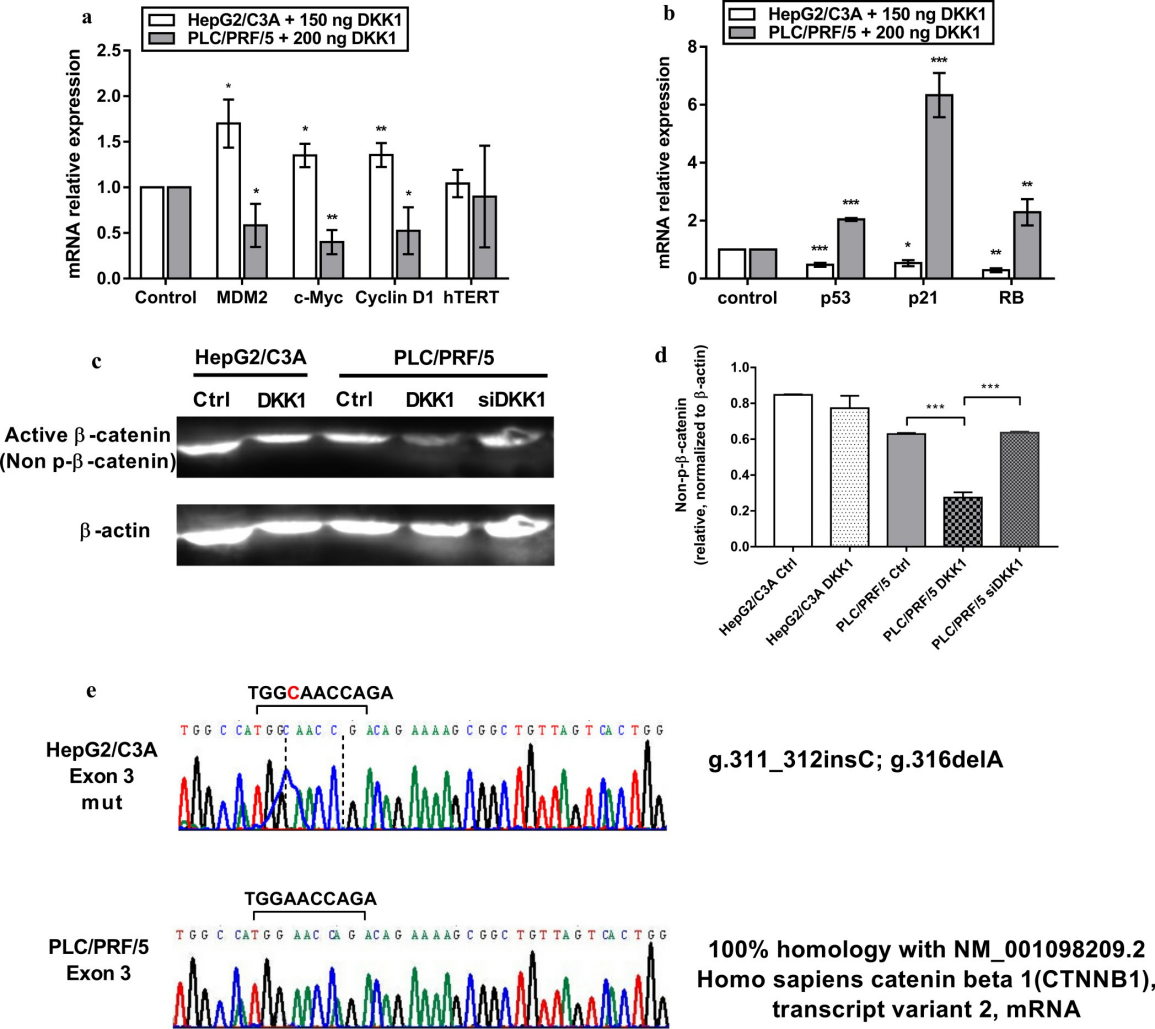
DKK1 induces opposite effects on the Wnt/beta-catenin pathway and on its target genes as a result of mutations in the *CTNNB1* gene. **(a)** mRNA expression of oncogenes c-Myc, cyclin D1, MDM2, and hTERT were decreased in PLC/PRF/5 cells but increased in HepG2/C3A cells after DKK1 treatment. **(b)** P53, P21 and RB transcription decreased after DKK1 treatment of HepG2/C3A cells but were increased in PLC/PRF/5 cells. mRNA expression was measured with qPCR, and the expression was normalized to that of GAPDH and expressed as the mean ± SD of triplicates. Western blot **(c)** and densitometric analysis to calculate the normalized ratio of non-phosphorylated β-catenin (active) to β-actin **(d)** protein expression. Data are expressed as the mean ± SD from 3 different experiments. * p <0.05, ** p <0.01, *** p <0.001, as indicated. **(e)** Sanger sequencing chromatograms of exon 3 of the *CTNNB1* gene shows point mutations in the HepG2/C3A cell line (g.311_312insC; g.316delA) while in PLC/PRF/5, no mutations were detected. The chromatograms were generated using ChromasPro software.

Since c-Myc and Cyclin D1 are direct targets for transcriptional regulation by the non-phosphorylated ẞ-catenin (active form), we supposed that the significant reduction of their expression in PLC/PRF/5 cells was related to the inhibition of the Wnt/ ẞ-catenin canonical pathway. We, therefore, examined protein levels of active ẞ-catenin following DKK1 treatment in both cell lines (Fig 2C and 2D). As expected, DKK1 treatment reduced active ẞ-catenin levels, while DKK1 downregulation with siRNA allowed active ẞ-catenin expression to rise back to control levels in PLC/PRF/5. In the HepG2/C3A cell line, DKK1 did not affect the active beta-catenin levels, and this inability of DKK1 to inhibit the Wnt/pathway might be explained by the possibility that the HepG2/C3A cell line harbors mutations in beta-catenin phosphorylation sites, thus impairing its degradation.

We, therefore, amplified and sequenced exon 3 and 4 of the *CTNNB1* gene, which carry the serine/threonine phosphorylation sites of ẞ-catenin. The DNA sequences of the chromatograms (Fig 2E) produced were BLAST searched (National Institutes of Health [NIH]) to identify any homology between these respective target sequences and those in GenBank (NIH). Two point mutations were revealed, **g.311_312insC** and **g.316delA** in exon 3 of the HepG2/C3A cell line, whereas exon 4 was devoid of any mutation in both cell lines (S2 Fig).

### DKK1 promotes inflammatory cytokines secretion in hepatocellular carcinoma cell lines

**Fig 3:**
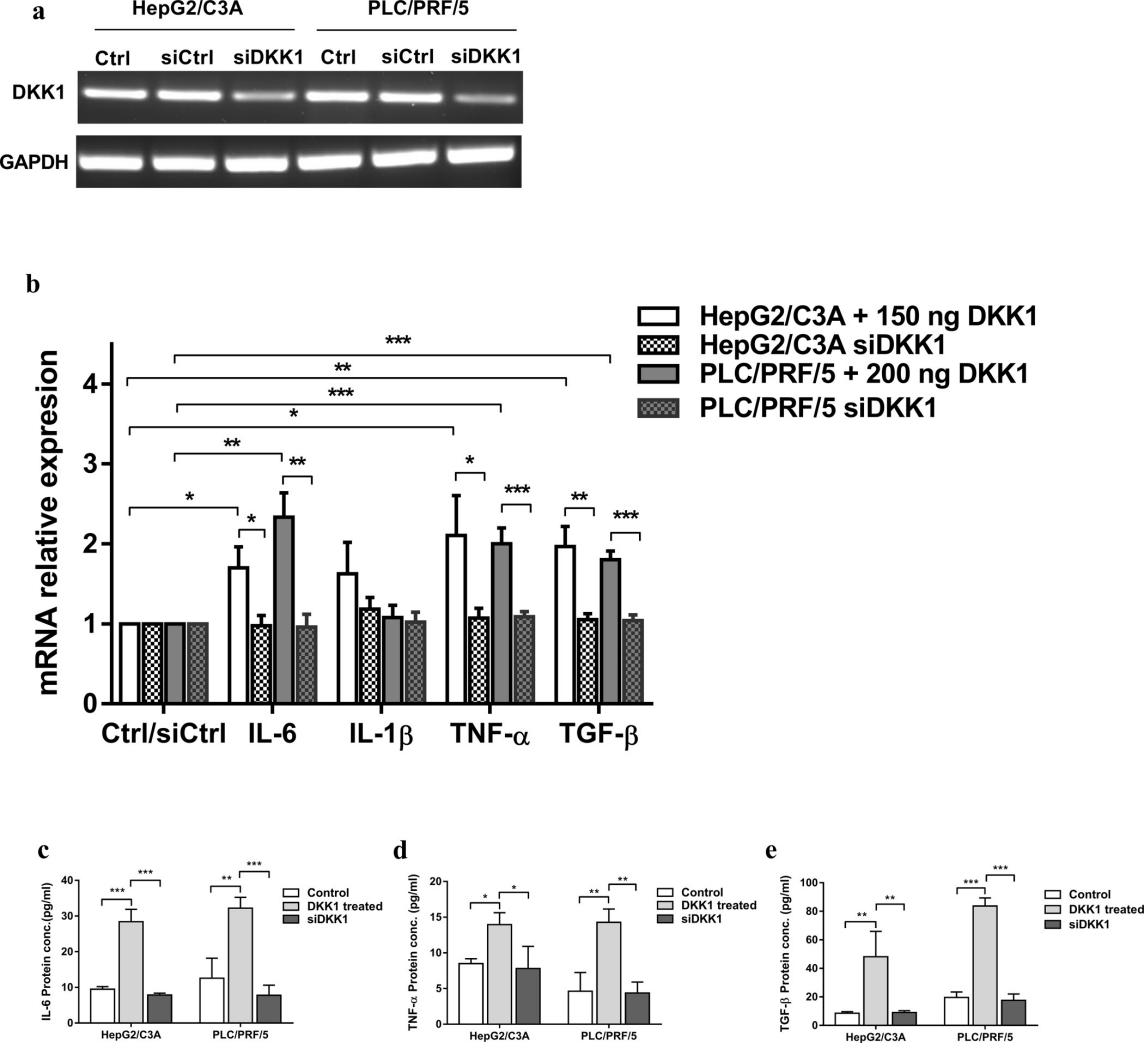
DKK1 promotes inflammatory cytokines secretion in hepatocellular carcinoma cell lines. **(a)** DKK1 knockdown was confirmed by PCR and qPCR (S.2). **(b)** Relative mRNA expression of IL-6, IL-1ẞ, TNF-α, and TGF-β1 after PLC/PRF/5 and HepG2/C3A cells were exposed to DKK1 for 48 h and 72 h, respectively, and following DKK1 knockdown. Expression was measured with qPCR and normalized to that of GAPDH. **(c, d, e)** IL-6, TNF-α and TGF-β1 secretion levels in the cell supernatants were measured after DKK1 treatment and in the absence of DKK1 expression by sandwich ELISA. Data are expressed as the mean ± SD of triplicates. * p <0.05, ** p <0.01, *** p <0.001, as indicated.

To measure the effect of DKK1 on the inflammation that accompanies tumor progression, qPCR and ELISA were performed to determine the expression levels of the proinflammatory cytokines TNF-α, IL-6, and IL-1ẞ after DKK1 treatment. TGF-β1, which, according to Fransvea et al. promotes HCC invasion and metastasis, was also evaluated [29]. Fig 3 clearly shows that recombinant DKK1 exacerbates inflammation and causes a significant increase in IL-6 at both the mRNA (HepG2/C3A (1.7-fold) (*p* < 0.05) and PLC/PRF/5 (2.33-fold) (*p* < 0.01)) and protein levels (increase of approximately 200% in HepG2/C3A and of 150% in PLC/PRF/5 DKK1 treated cells). There was also a significant increase in the mRNA (2.1-fold in HepG2/C3A and 2-fold in PLC/PRF/5) and protein levels (64% increase in HepG2/C3A and 200% in PLC/PRF/5) of TNF-α after DKK1 treatment.

Similarly, DKK1 induced a very significant increase (*p* <0.01) in the expression of TGF-β1 (Fig 3B) in both liver cancer cell lines with a very pronounced increase in TGF-β1 secretion of more than 300% (Fig 3E). However, the mRNA level of IL-1ẞ did not exhibit any substantial differences.

### DKK1 treatment enhances cell migration and invasion while its knockdown does the reverse

**Fig 4:**
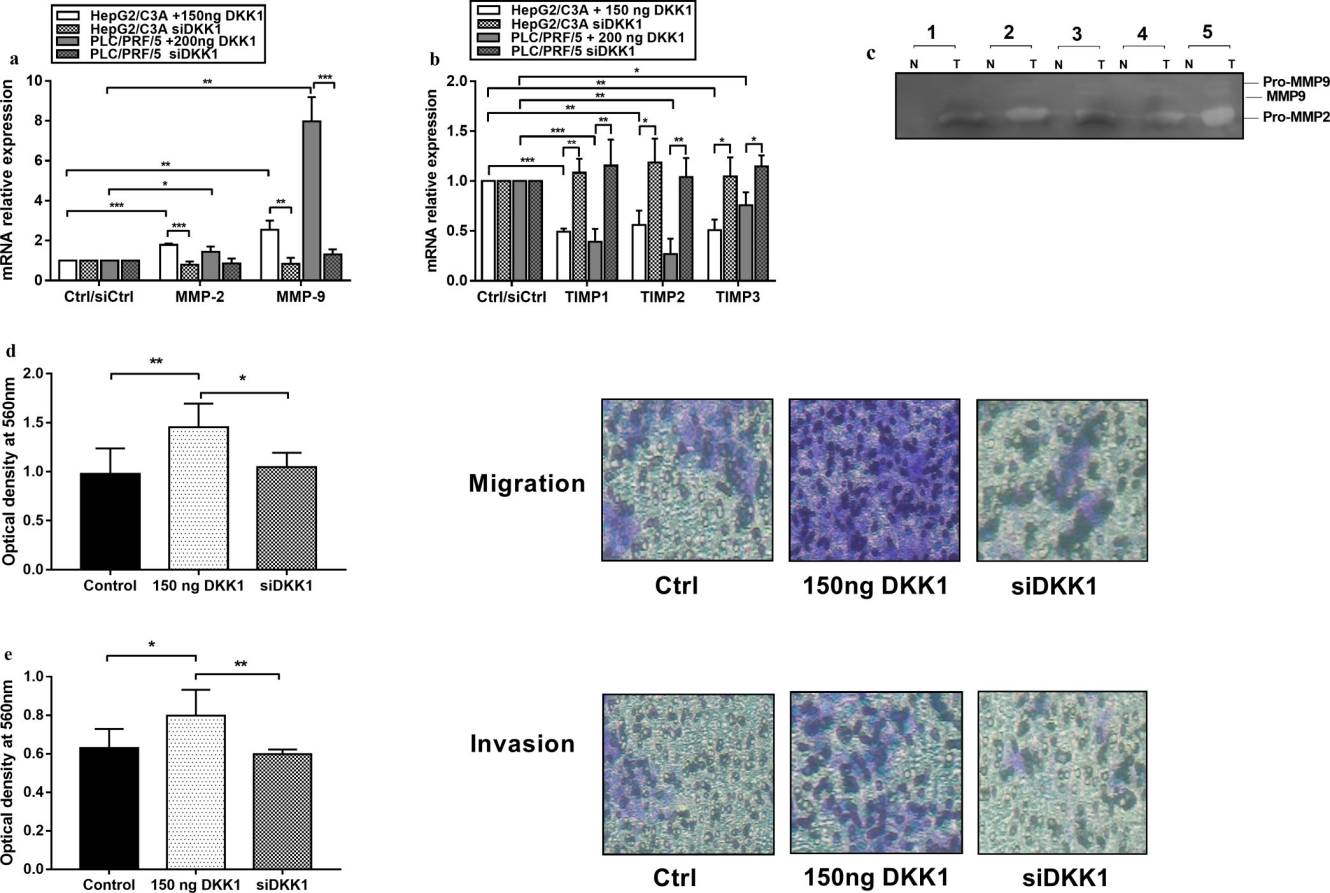
Recombinant DKK1 promotes the invasion and migration of hepatocellular carcinoma cell lines through shifting the MMPs/TIMPs ratio in favor of MMPs. **(a)** Relative mRNA expression levels of MMP-2 and MMP-9 show an overall significant increase after PLC/PRF/5 and HepG2/C3A cells were exposed to DKK1 for 48 h and 72 h, respectively, but this was reversed when DKK1 was silenced. **(b)** Relative mRNA expression levels of TIMP-1, TIMP-2, and TIMP-3 showing an overall significant decrease after DKK1 treatment and an increase after DKK1 knockdown. **(c)** Zymography for detecting MMP activity in the supernatants from HepG2/C3A (lanes 1–3) and PLC/PRF/5 (lanes 4 and 5); *T*, treated with 150 ng/ml and 200 ng/ml recombinant DKK1, respectively; *N*, untreated cancer cells. An obvious increase occurred in the latent form of MMP-2 in response to DKK1. Both the latent and active forms of MMP-9 were observed in the treated cells. **(d)** Migration assay of HepG2/C3A by Transwell Boyden chamber in the presence of 150 ng/ml recombinant DKK1 and in the absence of DKK1 expression. Migratory cells on the bottom of the polycarbonate membrane were stained and quantified based on the OD at 560 nm. Representative photographs of migratory HepG2. **(e)** Invasion of HepG2/C3A cells treated with 150 ng/ml recombinant DKK1, and DKK1 knock down cells assessed by the Transwell Boyden chamber. Invasive cells on the bottom of the invasion membrane were stained and quantified based on the OD at 560 nm. Representative photographs of invasive HepG2. The data are representative of at least 3 independent experiments and are expressed as the mean ± SD. * p <0.05, ** p <0.01, *** p <0.001, as indicated.

**Fig 5:**
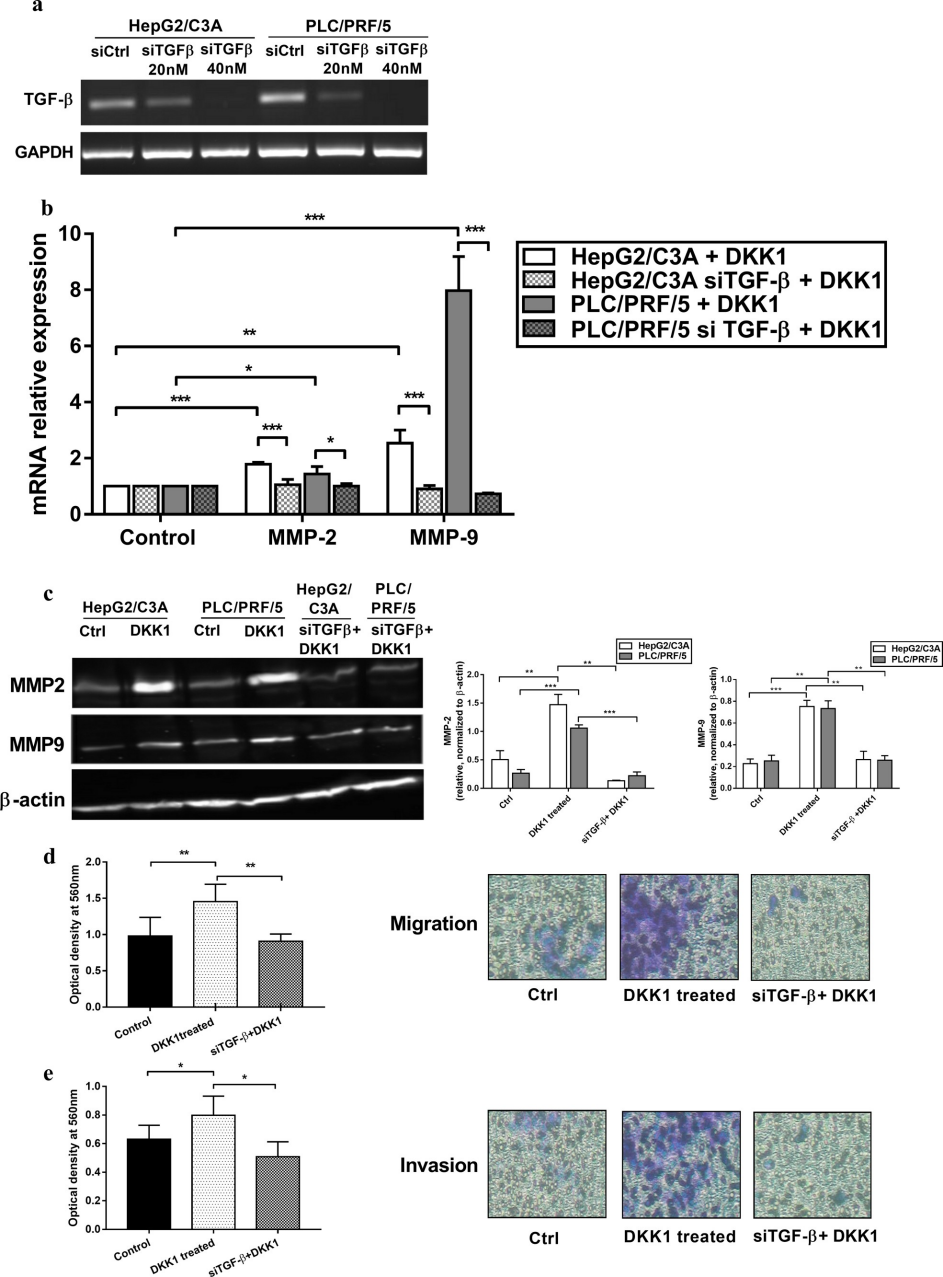
TGFB1 knockdown suppresses the DKK1 proinvasive effect. **(a)** TGF-β1 knockdown was confirmed by PCR and qPCR (S. 2). **(b)** Relative mRNA expression of MMP-2 and MMP-9 following DKK1 treatment in the presence or the absence of TGF-β1 expression (siTGFB1). Expression was measured with qPCR and normalized to that of GAPDH. **(c)** Western blot and densitometry analysis to calculate the normalized ratio of MMP-2 and MMP-9 to β-actin protein expression. Data are expressed as the mean ± SD from 3 different experiments. The siTGFB1 effects on DKK1 induced migration **(d)** and invasion **(e)** were assessed by the Transwell Boyden chamber assay. Representative photographs of migratory **(d)** and invasive **(e)** cells. All of the experiments were repeated 3 times. Data are expressed as the mean ± SD of triplicates. * p <0.05, ** p <0.01, *** p <0.001, as indicated.

The gene expression levels of MMP-2, MMP-9, TIMP-1, TIMP-2, and TIMP-3 were measured by qPCR in HCC cells treated with DKK1 solutions and in cells transfected with DKK1 siRNA. The transcription of MMP-2 was significantly increased in DKK1-treated HepG2/C3A cells (1.79-fold) (*p* <0.001) and DKK1-treated PLC/PRF/5 cells (1.43-fold) (*p* < 0.05) (Fig 4A). A more pronounced increase was observed in the transcription levels of MMP-9 in both cell lines (7.74-fold in PLC/PRF/5 and 2.5-fold in HepG2/C3A) (*p* <0.01) (Fig 4A). The TIMPs expression showed a significant overall decrease in both cell lines treated with DKK1 (Fig 4B). Western blot analysis showed an increase in MMP-2 and MMP-9 levels upon DKK1 treatment, in concordance with what had been observed at the mRNA level (Fig 5C).

To investigate whether recombinant DKK1 increases the proteolytic activity of MMP-2 and MMP-9, gelatin zymography was performed after HepG2/C3A and PLC/PRF/5 were treated with 150 ng/ml and 200 ng/ml recombinant DKK1, respectively. This technique identifies gelatinases in biological samples using SDS polyacrylamide gels containing gelatin and is appropriate for the detection of both the active and latent forms of the enzymes. Consistent with the observed increase in the gene expression of MMPs, recombinant DKK1 strongly induced gelatinolytic activity in both cell types with a noticeable increase in MMP-2 latent-form enzyme activity; the pro and activated forms of MMP-9 were also detected (Fig 4C).

The increase in MMP expression and gelatinolytic activity upon DKK1 treatment suggests that DKK1 influences the cellular machinery that mediates migration and invasion. Therefore, we evaluated the effect of recombinant DKK1 treatment on the invasive and migratory behavior of HCC cells using a Boyden chamber assay. The results indicated that in nontreated cells and in those transfected with siDKK1, the numbers of cells passing through the membrane were fewer in comparison to DKK1 treated cells. As shown in Fig 4D, the migratory potential increased significantly (48.3%) (p < 0.01) after DKK1 treatment and decreased significantly after DKK1 knockdown (27.9%) (p<0.05). Fig 4E shows that the invasive potential of HCC cells similarly increased significantly (26.67%) (p < 0.05) when compared to untreated cells and decreased in transfected cells when compared to treated cells (25.14%) (p<0.01). Consistent with the zymography, qPCR and Western blot results measuring the expression levels of invasion-related genes, recombinant DKK1 promotes the invasion of hepatocellular carcinoma cell lines.

### TGF-β1 silencing suppresses the DKK1 proinvasive effect

TGF-β has been reported to contribute to tumor progression by inducing epithelial-to-mesenchymal transition, cell migration and invasion of epithelial tumor cells through stimulating MMP-2 and MMP-9 secretion into the tumor microenvironment [30]. The significant increase in TGF-β1 mRNA and protein expression in both cell types following DKK1 treatment led us to examine whether TGF-β1 may act as a potential mediator in DKK1 stimulated cell invasion. Thus, a TGF-β1 knockdown was performed (Fig 5A). TGF-β1 siRNA abolished DKK1 induced invasion and migration in both HepG2/C3A and PLC/PRF/5 cells. MMP-2 and MMP-9 gene and protein expression were both negatively affected by TGFB1 knockdown (Fig 5B and 5C). In fact, upon treatment with recombinant DKK1, cells transfected with siTGFB1 showed a very significant decrease (p<0.001) in MMP-9 gene expression when compared to non-transfected cells (10.6-fold decrease in PLC/PRF/5 and 2.78-fold decrease in HepG2/C3A). A less pronounced decrease was observed in MMP-2 gene expression (1.9-fold decrease in PLC/PRF/5 and 1.7-fold decrease in HepG2/C3A). Accordingly, western blot analysis revealed that MMP-2 and MMP-9 protein levels were reduced upon knockdown of TGF-β1 in both cell lines (Fig 5C). In all cases transfection with siRNAs had no effect on the protein levels of the internal control (β-ACTIN). Our data suggest that TGF-β1 is essential for the cell migration and invasion induced by DKK1 in hepatocellular carcinoma cell lines.

## Discussion

With nearly 1 million cases diagnosed each year, HCC is considered to be the most common primary liver tumor in the world, most frequently occurring in livers that undergo a chronic inflammatory process that induces continuous cycles of regeneration-necrosis [31]. Many genetic alterations accumulate during hepatocarcinogenesis. These alterations affect several signaling pathways, the deregulation of which causes uncontrolled proliferation, a decrease in apoptosis and increased invasion and angiogenesis [32]. The Wnt/beta-catenin pathway is considered to be the most frequently activated oncogenic pathway in HCC, contributing significantly to its initiation, growth, and metastasis. The study of this pathway is relevant to better understanding the hepatocarcinogenesis. We have previously suggested that treatment of hepatocellular carcinoma cell lines with recombinant "DKK1", a negative regulator of the Wnt pathway, may have an antitumor effect similar to that obtained with Pimozide, which acts by inhibiting the Wnt/beta-catenin pathway to reduce the proliferation of HepG2 and Hep3B lines according to V, Fako et al. [33].

Indeed, activation of the Wnt pathway induces the upregulation of the oncoprotein c-Myc, which is known to be a DKK1 repressor [34]. This upregulation generates a positive feedback loop and thus further amplifies the activation of canonical Wnt signaling. V, H, Cowling et al. attempted to overcome this repression by transfecting MDA-MB-231 and T-47D mammary tumor cell lines with a DKK1 carrier vector [35]. This manipulation resulted in a reduction in the proliferation of both cell types with a remarkable extension of the cell doubling time [35]. In that context, we thought it would be interesting to treat hepatic cancerous lines with the recombinant protein DKK1 to study its effect on tumor progression.

Our results showed that DKK1 does not have an antiproliferative effect on our liver cancer cell lines. This finding may be explained by the fact that the activation of the Wnt/beta-catenin pathway in HepG2/C3A cells and PLC/PRF/5 cells is not attributed to a decrease in DKK1 expression but, rather, to mutations in the cellular components of this pathway, including beta-catenin, AXIN and APC, rendering beta-catenin degradation impossible and increasing its translocation to the nucleus and the subsequent activation of target genes involved in proliferation and tumor invasion. Koch et al. described mutations in the degradation box of beta-catenin that prevent its degradation in HepG2 cells, which is further evidence to support our proposed explanation [36]. On the other hand, A, M, Mikheev et al. demonstrated that the ectopic expression of DKK1 mediated by retroviral vectors in HeLa cells led to a significant inhibition of anchorage-independent growth. In addition, the same group demonstrated that the inhibition of tumorigenicity by DKK1 was not related to the inhibition of beta-catenin-dependent transcription [37]. We therefore presume that the effect of DKK1 on cell proliferation is associated with a pathway other than the Wnt/beta-catenin pathway.

Our results also showed that treatment with different concentrations of DKK1 did not have a significant effect on AFP secretion in HepG2/C3A cells at 24 hrs. The decrease observed at 48 hrs cannot be related to DKK1 since according to the ATCC, HepG2/C3A cells will decrease AFP secretion once they reach confluency. However, we noticed a significant increase in AFP mRNA expression after DKK1 treatment. In fact, AFP expression is regulated by several factors, including p53 and HNF-3 (hepatocyte nuclear factor 3), which bind to the same site in a mutually exclusive manner. HNF-3 protein activates while p53 represses the gene expression of AFP. KC, Lee et al. revealed a correlation between p53 activation and alpha-fetoprotein suppression [38]. Given the results of qPCR in this study, which indicate a significant decrease in p53 expression by HepG2/C3A cells following treatment with DKK1, one would predict an increase in AFP expression. In the PLC/PRF/5 cells, there was an increase in both the expression and secretion of AFP. At the same time, we noticed an increase in the expression of p53, a repressor of AFP expression. This finding can be explained by the fact that AFP expression is regulated by factors other than just p53. In fact, HNF1 is a transcription factor that binds to the promoter region of AFP and activates its transcription. The expression of HNF1 is regulated mainly by HNF4-α (hepatocyte nuclear factor 4 alpha), the expression of which is induced by TGF-β [39]. Therefore, the significant increase in TGF-β expression observed in PLC/PRF/5 explains the increase in AFP expression and secretion in the absence of a decrease in p53 expression.

Regarding the expression of tumor suppressor genes and oncogenes, the results were discordant between the two cell types. A significant decrease in the expression of p53, p21, and RB and an increase in the expression of the negative regulator of p53, MDM2, as well as c-Myc, Cyclin D1 and hTERT were observed in HepG2/C3A. These results support an oncogenic role of DKK1 in HepG2/C3A. Western blot analysis showed that DKK1 treatment had no significant effect on beta catenin active levels in HepG2/C3A. This tumor-promoting effect may thus be related to the activation by DKK1 of beta-catenin-independent pathways. In fact, the lack of an effect on the Wnt/beta-catenin pathway in HepG2/C3A could be explained by the mutations found in exon 3 since exon 3 is known to encode serine-threonine phosphorylation sites for GSK-3ẞ and casein kinase-1 that activate beta-catenin degradation [40]. Even in the presence of the canonical pathway inhibitor DKK1, such mutations will stabilize beta catenin through abrogation of its degradation.

However, in PLC/PRF/5, the significant increase in p53, p21 and RB expression and the significant decrease in c-Myc, Cyclin D1 and MDM2 expression suggest a tumor suppressor effect of DKK1 on this cancer cell line through inhibition of the canonical pathway. This inhibition was mediated by a decrease in ẞ-catenin active levels, hence explaining the downregulation of ẞ-catenin target genes expression, mainly c-Myc and cyclin D1, upon DKK1 treatment. Indeed, Mi Hee Kwack et al. showed that the transfection of plasmids carrying the DKK1 gene into a clone of the HCC cell line SNU-475 led to an inhibition of tumorigenicity [41]. H, Pang et al. have shown that in the case of small-cell lung cancer, introducing a DKK1 expression vector into SBC-3 cells leads to an increase in tumorigenicity [42].

The inverse effects shown in our tests on HepG2/C3A and PLC/PRF/5 reflect differences in the genetic components of HCC between these two liver cancer cell lines. Those differences were confirmed by Sanger sequencing. Given the established link between chronic inflammation and different stages of tumor development, and considering the results of murine studies indicating that aberrant activation of the Wnt pathway generates a proinflammatory process, we deemed it urgent to study the effect of an inhibitory molecule of this pathway on the main inflammatory cytokines IL-1ẞ, IL-6 and TNF-α [43]. Beta-catenin induces an intrinsic inflammatory transcriptional program by upregulating chemokines and proinflammatory cytokines, such as Ccl2, Ccl5, IL-15 and IL-18 [43]. Cytokines produced by cancer cells act to create optimal growth conditions in the tumor microenvironment. In addition, a decrease in oxygen availability causes an increase in the expression of the cytokines IL-1ẞ, IL-6 and TNF-α, which in turn promotes tumor progression by stimulating VEGF expression [44]. On the other hand, IL-1ẞ can stimulate the expression of metastatic genes such as MMPs [44]. Our results showed that DKK1 induces an increase in the expression and secretion of the proinflammatory cytokines IL-6 and TNF-α in both PLC/PRF/5 and HepG2/C3A, but the mechanisms involved are poorly understood. We were also interested in studying the effect of DKK1 on TGF-β expression since it has been shown that TGF-β is capable of inducing EMT, thereby facilitating cancer dissemination [45,46]. The results showed a significant elevation of TGF-β expression and secretion in the tumor microenvironment (TME) following treatment with the recombinant DKK1 protein. This increase promoted the migration and invasion process. These data support the hypothesis that DKK1 induces the secretion of several factors by tumor cells, thereby remodeling the TME in such a way as to promote tumorigenesis and eventually metastasis.

Tumor invasion represents an important turning point in the evolution of HCC and has a profound impact on the vital prognosis, significantly reducing the chances of recovery. A major group of proteases that has been implicated in tumor invasion and metastatic progression is the matrix metalloproteinases (MMPs). In fact, the epithelial-mesenchymal transition process, which allows cancer cells to undergo dedifferentiation and to acquire migratory and invasive capacities, has been shown to be induced by the overexpression of MMPs, whose activity is modulated by the endogenous tissue inhibitors of metalloproteinases (TIMPs) [47]. To test the hypothesis that DKK1 promotes tumor invasion, we performed gelatin zymography, western blot and qPCR to assess the expression pattern of gelatinases (MMP-2, MMP-9). Our data demonstrated that in both cell types, DKK1 increased gelatinolytic activity and the mRNA expression of MMP-9 and MMP-2 while decreasing the mRNA expression of TIMPs. These findings support a role for DKK1 as a promoter of tumor progression and invasion in HCC. To further confirm this hypothesis, a Boyden chamber assay was performed and the results showed that DKK1 promotes cell invasion and migration *in vitro*. In reverse, when DKK1 was silenced, tumor invasion was significantly reduced. Interestingly, several years ago, Chen et al. disclosed that DKK1 promotes HCC cell migration and invasion and that it exerted its pro invasive function by stimulating beta catenin expression and transcription, although our results suggested that DKK1 downregulates beta catenin expression in PLC/PRF/5 and still induces invasion and migration in this cell line. Therefore, we suggest that the pro invasive function of DKK1 passes through another pathway and is independent of the Wnt/ẞ-catenin pathway.

We showed previously that TGF-β is highly induced by DKK1 treatment. In fact, several studies have indicated that TGF-β can induce invasive and migratory phenotypes in cancer cells and can act through autocrine signaling to promote EMT by up-regulating MMPs [48,49]. Therefore, the effect of DKK1 on cell migration and invasion and on MMPs secretion was determined in the absence of TGF-β expression in tumor cells. Interestingly, our results indicated that the DKK1 proinvasive effect on HCC cell lines was abrogated in tumor cells lacking TGF-β expression. Thus, inhibiting TGF-β can decrease DKK1 induced invasion and migration.

## Conclusion

In conclusion, the results of this study suggest that recombinant DKK1 facilitates tumor invasion and migration through remodeling the tumor microenvironment and inducing the secretion of proinflammatory cytokines. Interestingly, TGF-β appears to mediate the DKK1 proinvasive effect, endorsing the relevance of TGF-β in HCC progression and its potential as a therapeutic target. As for oncogenes and tumor suppressor genes, DKK1 appears to generate opposite effects due to mutations in beta-catenin that compromise the ability of DKK1 to inhibit the canonical Wnt pathway.

## Supporting information

S1 Table List of primers used in this paper. (DOCX)

S1 Fig **qPCR analysis of DKK1 (a) and TGF-ẞ1 (b) gene expression in hepatocellular carcinoma cell lines HepG2/C3A and PLC/PRF/5 after siRNA transfection.** GAPDH was used as an internal control. Data are expressed as the mean ± SD of triplicates. * p <0.05, ** p <0.01, *** p <0.001, as indicated.
(TIF)

S2 Fig Sequence chromatograms of the exon 4 of *CTNNB1* gene in HepG2/C3A and PLC/PRF/5 showing no mutation. The chromatogram was generated using ChromasPro software. Sequences of the chromatograms were BLAST searched (National Institutes of Health [NIH]) to identify homology between these respective target sequences and those in GenBank (NIH).
(TIF)

S3 Fig Effect of DKK1 on cellular proliferation of hepatocellular carcinoma cell lines. HepG2/C3A and PLC/PRF/5 cells were seeded at a density of 10^3^ cells /well and then treated with different concentrations of DKK1 for 24 h, 48 h or 72 h. The proliferation rate was measured using the WST8 test and calculated from the absorbance values **(a, b, c)**
(TIF)
