# Supplementary material for: DKK1 promotes hepatocellular carcinoma inflammation, migration and invasion: Implication of TGF-β1
Source: PLoS One. 2019 Sep 30;14(9):e0223252. doi: 10.1371/journal.pone.0223252 (PMC6768474; doi:10.1371/journal.pone.0223252)
Supplement: S1 Table — (DOCX) [file pone.0223252.s001.docx]

**S1 Table. List of primers used in this paper**

| Primers | Forward | Reverse |
| --- | --- | --- |
| DKK1 | 5´-GTGCAAATCTGTCTCGCCTG-3´ | 5´-GCACAACACAATCCTGAGGC-3´ |
| CTNNB1 Exon3 | 5´-AGTAACATTTCCAATCTACTAATGC-3´ | 5´- CAAAACTGCATTCTGACTTTCA-3´ |
| CTNNB1 Exon4 | 5´-ATGCTGAACTGTGGATAGTGA-3´ | 5´- GAGCATTTACTTCAAAGCAGAC-3´ |
| IL-1ẞ | 5´-CAGAAGTACCTGAGCTCGCC-3´ | 5´-AGATTCGTAGCTGGATGCCGA-3´ |
| IL-6 | 5´-GGTACATCCTCGACGGCATCT-3´ | 5´-GTGCCTCTTTGCTGCTTTCAC-3´ |
| TIMP-1 | 5´-GACCAAGATGTATAAAGGGTTA-3´ | 5´-GAAGTATCCGCAGACACTCTCCAT-3´ |
| TIMP-2 | 5´-AGGCGTTTTGCAATGCAGAT-3´ | 5´-TCCAGAGTCCACTTCCTTCTCACT-3´ |
| TIMP-3 | 5´-CAGGACGCCTTCTGCAACTC-3´ | 5´-AGCTTCTTCCCCACCACCTT-3´ |
| MMP-2 | 5´-ATATACCATGATCTACAGGAACTTGGTAGT-3´ | 5´-GGTGACACCTGTTCTCACTCACA-3´ |
| MMP-9 | 5´-GCTTCTACTGGCGCGTGAGT-3´ | 5´-CATAGGTCACGTAGCCCACCTG-3´ |
| TGF-ẞ | 5´-CGCGTGCTAATGGTGGAAA-3´ | 5´-GTGTGTACTCTGCTTGAACTTTCA-3´ |
| TNF-α | 5´-GGTATGAGCCCATCTATCTG-3´ | 5´-TTTTTGAGCCAGAAGAGGTT-3´ |
| AFP | 5´-CAGCCACTTGTTGCCAACTC-3´ | 5´-GGCCAACACCAGGGTTTACT-3´ |
| Cyclin D1 | 5´- CCTTGAGGGACGCTTTGTCT-3´ | 5´- GCCTTTGGCCTCTCGATACA-3´ |
| hTERT | 5´-GACGTAGTCCATGTTCACAATCG-3´ | 5´-CGTCCAGACTCCGCTTCATC-3´ |
| P53 | 5´-CAAGCAATGGATGATTTGATGCT-3´ | 5´-TGGGTCTTCAGTGAACCATTGT-3´ |
| P21 | 5´-CCGAAGTCAGTTCCTTGTGGAG-3´ | 5´-CACCTGTGAACGCAGCACAC-3´ |
| RB | 5´-GCAAATTGGAAAGGACATGTGA-3´ | 5´-GAAACTTTTAGCACCAATGCAGAA-3´ |
| MDM2 | 5´-ATATACCATGATCTACAGGAACTTGGTAGT-3´ | 5´-GGTGACACCTGTTCTCACTCACA-3´ |
| c-Myc | 5´-CACCACCAGCAGCGACTCT-3´ | 5´-TTCCACAGAAACAACATCGATTTC-3´ |
| GAPDH | 5´-GCACCACCAACTGCTTAGCA-3´ | 5´-CTTCCACGATACCAAAGTTGTCAT-3´ |
