# Supplementary figures and images for: DKK1 promotes hepatocellular carcinoma inflammation, migration and invasion: Implication of TGF-β1
Source: PLoS One. 2019 Sep 30;14(9):e0223252. doi: 10.1371/journal.pone.0223252 (PMC6768474; doi:10.1371/journal.pone.0223252)

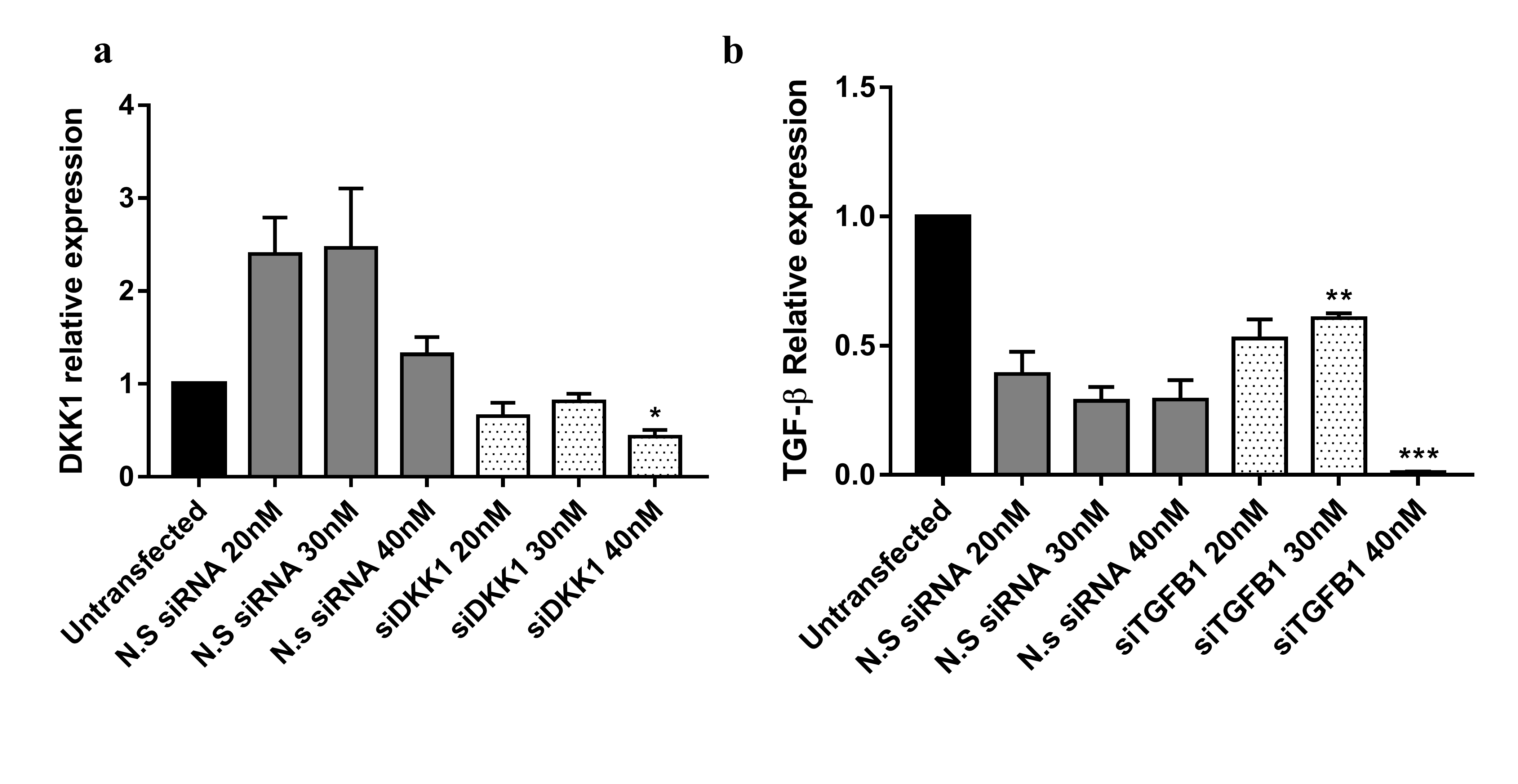

Supplement: S1 Fig — qPCR analysis of DKK1 (a) and TGF-ẞ1 (b) gene expression in hepatocellular carcinoma cell lines HepG2/C3A and PLC/PRF/5 after siRNA transfection. GAPDH was used as an internal control. Data are expressed as the mean ± SD of triplicates. * p <0.05, ** p <0.01, *** p <0.001, as indicated. (TIF) [file pone.0223252.s002.tif]

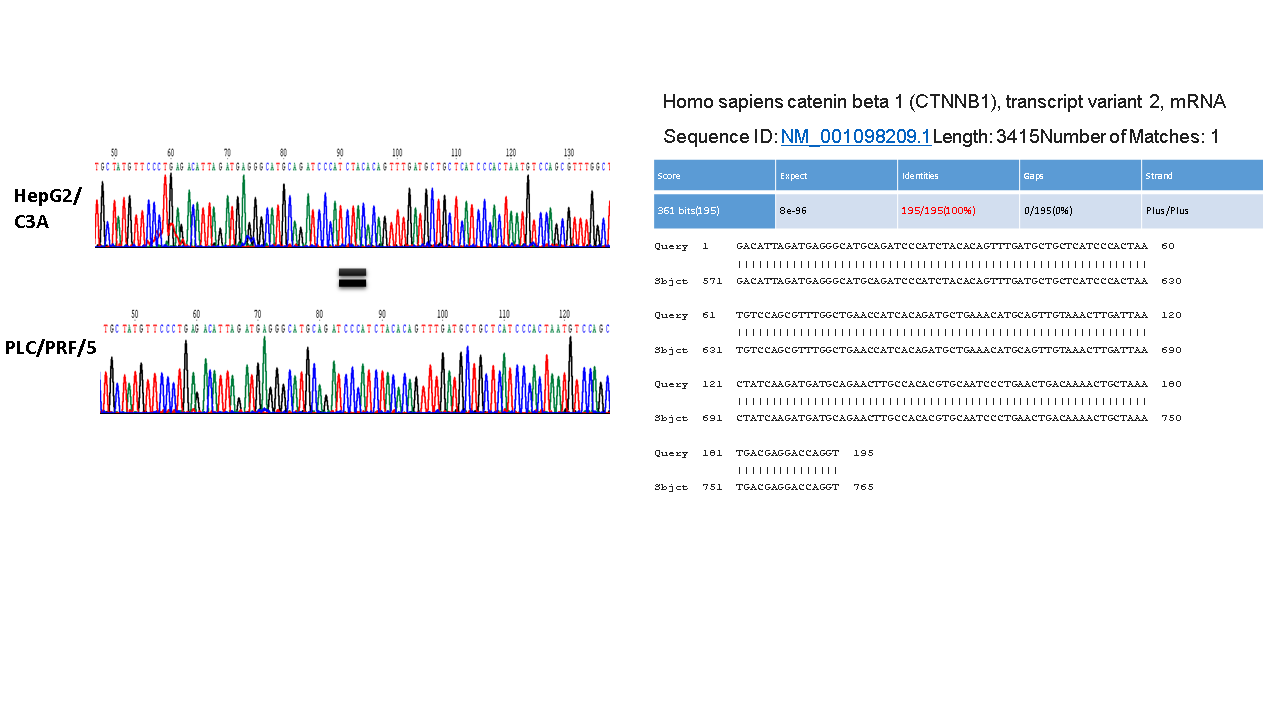

Supplement: S2 Fig — The chromatogram was generated using ChromasPro software. Sequences of the chromatograms were BLAST searched (National Institutes of Health [NIH]) to identify homology between these respective target sequences and those in GenBank (NIH). (TIF) [file pone.0223252.s003.tif]

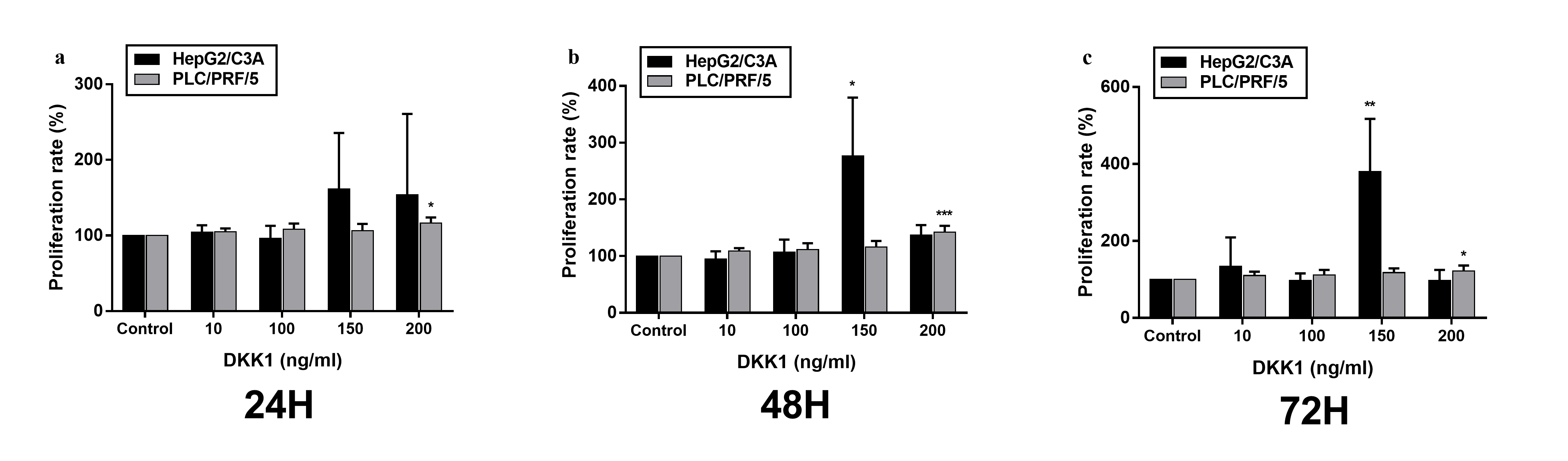

Supplement: S3 Fig — HepG2/C3A and PLC/PRF/5 cells were seeded at a density of 103 cells /well and then treated with different concentrations of DKK1 for 24 h, 48 h or 72 h. The proliferation rate was measured using the WST8 test and calculated from the absorbance values (a, b, c) (TIF) [file pone.0223252.s004.tif]
